# Aggregation Site Choice by Gregarious Nymphs of the Desert Locust, *Schistocerca gregaria*, in the Sahara Desert of Mauritania

**DOI:** 10.3390/insects9030099

**Published:** 2018-08-13

**Authors:** Koutaro Ould Maeno, Mohamed Abdallahi Ould Babah Ebbe

**Affiliations:** 1 Japan International Research Center for Agricultural Sciences (JIRCAS), Livestock and Environment Division, Ohwashi 1-1, Tsukuba, Ibaraki 305-8686, Japan; 2 The Mauritanian Desert Locust Centre: Centre National de Lutte Antiacridienne (CNLA), Nouakchott, BP 665, Mauritania; maouldbabah@yahoo.fr; 3 Institut du Sahel (INSAH)/CILSS, BP 1530 Bamako, Mali

**Keywords:** aggregation, migration, night roosting site, plant community, phase polyphenism, *Schistocerca gregaria*

## Abstract

Animals often aggregate at certain sites during vulnerable periods such as night-roosting as an anti-predatory strategy. Some migratory gregarious animals must regularly find new night-roosting sites, but how they synchronously choose such sites is poorly understood. We examined how gregarious nymphs of the desert locust, *Schistocerca gregaria* Forskål (Orthoptera: Acrididae), aggregate at certain plants for night-roosting in the Sahara Desert. Migratory bands of last instar nymphs climbed trees around dusk and roosted there overnight. A spatial autocorrelation analysis of plants indicated that the larger locust groups formed at the larger plants within the local plant community. Other large groups were not formed near the large tree, but smaller groups were patchily distributed. Plant height was the primary cue used by migratory bands to choose night-roosting plants. A nearest-neighbor distance analysis showed that single conspicuous large trees with scattered smaller plants were distributed locally. This plant community structure and negative geotactic ascending behavior of gregarious nymphs may force them to concentrate at the landmark plant from all directions and afar. This plant-size-dependent roosting site choice may contribute for developing artificial trapping systems for locusts and inciting to a new environment-friendly night control approach.

## 1. Introduction

Many animals aggregate with conspecifics at specific sites as an anti-predatory strategy [1,2]. Aggregation may reduce the probability of predation through dilution effects, and some aggregating sites also function as refuge against predators [3]. Individual attraction may lead conspecifics to aggregate [4] and species-specific microhabitat choice may jointly concentrate individuals in a limited area [5]. Although individual attraction has been extensively studied to elucidate the mechanism regulating aggregation, it is also necessary to understand species-specific microhabitat preferences [6].

Night is a regular vulnerable period for diurnal animals, because their escaping performance is reduced at night due to darkness and lowered temperature [7]. To overcome such a vulnerable period, some animals aggregate [3,7] and roost on protective site [8]. Sedentary animals can use same night-roosting site, while migratory animals have to change it depending on the various habitats encountered during migration. Such a flexible night-roosting site choice to satisfy the necessary refuge quality and aggregation space would be adaptive for migratory animals, but the primary cues to determine the timing and location of the new night-roosting site are poorly understood.

Locusts grasshopper species live alone or in extensive groups comprising millions of individuals and exhibit density-dependent phase polyphenism [9,10,11]. In the desert locust, *Schistocerca gregaria* (Forskål, 1775), solitarious locusts occurring at low density avoid each other and are sedentary, whereas gregarious locusts occurring at high density are attracted to each other and move long distances in a group [12]. Phase transition from solitarious phase to gregarious one occurs when solitarious locusts are crowded with conspecifics [10]. Aggregation has been regarded as a typical characteristic of the gregarious phase. Although collective movement has been extensively studied in locusts [10,11,13], the mechanism underlying aggregation formation is not known completely.

Some field observations and simulation models showed that a heterogeneous plant distribution promotes aggregation of nymphs, whereas a uniform distribution promotes scattering [12,14,15,16,17]. However, we frequently observed that dense aggregations occurred at certain night-roosting plants even within a uniform plant distribution in the field, so plant distribution alone cannot explain the aggregation formation. In *S. gregaria*, night-roosting plant preference influence on their distribution: solitarious nymphs and adults and gregarious adults selectively roost on relatively large plants during a night [18,19,20]. It is reasonable to assume that night-roosting plant preference of gregarious nymphs may lead aggregation. Accordingly, we hypothesized that gregarious nymphs were also attracted to larger plants as a night-roosting site and form dense aggregation there. This hypothesis should be tested in the field at a site where various sizes of plants co-occur within a plant community. During a field survey in one of the major breeding and recession areas of the desert locust in Mauritania, we encountered a suitable situation to test this hypothesis. We examined how gregarious nymphs of *S. gregaria* chose night-roosting plants and their level of aggregation to identify cue(s) of plant characteristics used for aggregation site choice.

Migratory bands of *S. gregaria* are comprised of various sizes of groups [12]. Although mutual attraction has been studied at an individual level [21], we have little understanding of this at the group level due to the difficulty of quantifying group distribution in the field [11]. Ellis and Ashall observed that migratory bands fused with each other when two bands crossed paths while marching [12]. If groups attract each other at the individual level within a local area, it can be predicted that a single large group would be formed with no corresponding larger groups nearby. However, little information is available about spatial distribution of different sizes of locusts groups. The process of aggregation formation in gregarious locusts can be investigated by observing night-roosting aggregation formation, because they roost on patchily distributed individual plants and do not descend to the ground until dawn [12]. Therefore, we hypothesized that migratory bands form a single large aggregation at the night-roosting site when their preferable night-roosting plants were available. We also examined whether negative geotaxis (i.e., ascending) plays a role in aggregation formation on the night-roosting plants, the locusts’ geotactic response to gravity was investigated at night. We also tested these hypotheses by surveying migratory bands of *S. gregaria* last instar nymphs in their natural habitat in the Sahara Desert. Our observations suggested how gregarious locusts choose the most appropriate night-roosting plants from among the local plant community and relief by responding to plant size and form a dense aggregation. We discuss the behavioral mechanism that sustains aggregation at the group level.

## 2. Materials and Methods

### 2.1. Study Animals

Gregarious nymphs of *S. gregaria* strongly aggregate in bands that can range from a few hundred to nearly 500,000 individuals [12]. Nymphs exhibit a stereotypic daily behavior pattern in winter. Most nymphs roost high in larger trees and bushes at night, descend to the ground at dawn, feed and march until around noon, hide in the shade or low plants during the hottest part of the day, march and feed again in the afternoon, then ascend nocturnal roosting plants around dusk [12]. During daytime migration, gregarious nymphs can march up to 1.0 km per day in dense bands [12]. This migration occurs at all developmental stages. Gregarious nymphs must therefore select a new night-roosting plant for each bout of migration during the nymphal stage.

**Figure 1:**
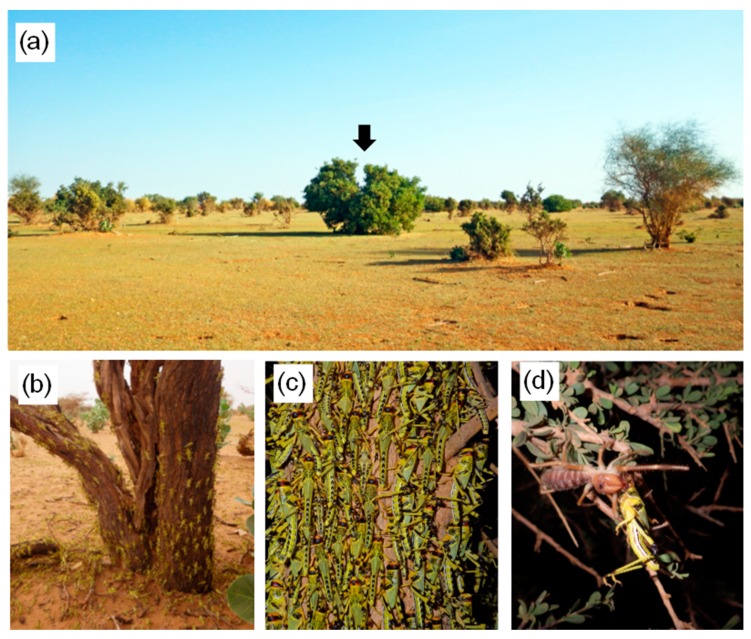
(**a**) Plant community used as a night-roosting site by gregarious nymphs of *Schistocerca gregaria*. Arrow indicates the largest tree roosted on by the largest locust group in the survey area. (**b**) Marched gregarious nymphs started climbing on a night-roosting tree around dusk. (**c**) Aggregation of gregarious nymphs on a night-roosting plant. (**d**) A camel spider (Solifugae) feeding on a *S. gregaria* nymph on the roosting plant.

Camel spiders (Solifugae), which actively wandered on the ground and plants, attacked roosting gregarious nymphs on the plants (Figure 1d). During one observation, three predations were observed. Each camel spider captured a single nymph and consumed the prey item there.

### 2.2. Study Area

The West African country of Mauritania is an important area where gregarization occurs within the recession zone of the desert locust [22,23]. The study site (5 km × 5 km; 19°23′ N, 14°35′ W) is located near Akjoujt in northwestern Mauritania. The area is a vast plain with a variety of soil types (rocky dry soils, dunes and playa) and vegetation types consisting of low-growing desert annuals (grasses, herbs, vines, etc.) (Figure 1a). We conducted field surveys from 23 to 26 November 2016 when we encountered migratory bands of last instar nymphs. We observed group oviposition in the middle of October and dark hatchlings typical characteristics of gregarious phase near the survey site at the beginning of the November. Based on these facts, the nymphs observed in this survey site were identified as being in the gregarious phase. Mean temperature was 24.9 °C (SE ± 0.1 °C, range 21.0–28.4 °C) and mean humidity was 38.1% (SE ± 0.3%, range 32.0–45.5%) during the daily observation period (21:00–07:00).

### 2.3. Sampling Regime

We followed different 10 marching migratory bands (>10,000 individuals each) of mostly last instar nymphs (with a small number of fourth instar nymphs) encountered within the survey site without disturbing them until they climbed night-roosting plants (17:00–19:00). The plant that the majority of a single band members (>60%) climbed at dusk was designated as the center at each survey site. To determine the relationship between the largest group (center) and other groups roosted on other individual plants, we recorded the group sizes, the distances from the center plant, and the plant size and species. Field surveys were conducted at night (21:00–07:00). Sunrise and sunset of local time were about 7:09 and 18:19, respectively.

### 2.4. Group Size and Group Interaction

Accurately counting the number of locusts roosted on the plants was difficult because there were too many and they were hidden under branches. We estimated the number of nymphs perching on each plant by direct counting after 20:00 when locusts rarely descended to the ground from the roosting plants and they did not move actively. Each group on an individual plant was categorized as one of six sizes: 0 (0 locusts), 1 (<10), 2 (10–100), 3 (>100–1000), 4 (>1000–10,000) and 5 (>10,000), based on the estimation method described by Maeno et al. [24].

Our preliminary observations since 2011 in Mauritania indicated that migratory bands formed the larger group on relatively large plants during a night. To determine the spatial interaction between the largest group and other groups roosting on plants, circular quadrats were circumscribed using the largest group as the center point and all groups within a 20 m radius (1256 m^2^) were surveyed. The distance between the center and the other individual groups was recorded by using a laser rangefinder (Bosch, DLE70, Stuttgart, Germany). For each night-roosting plant, the size (maximum width and height) and species were determined at the same time.

### 2.5. Plant Size and Plant Abundance

Plants are patchily distributed at the study site. *Calotropis procera* (Asclepiadaceae), *Acacia tortilis* (Fabaceae), *Maerua crassifolia* (Capparaceae), and *Boscia senegalensis* (Capparaceae) were frequently used as night-roosting plants.

To determine the size of plants at the survey site, the maximum length, width, and height were measured for individuals of the four dominant plant species by using a tape measure, according to Maeno et al. [18]. Plants less than 10 cm in any one of the dimensions were not measured. The volume (m^3^) of each plant was calculated as maximum length × width × height. Abundance of each plant species was calculated from the circular quadrats (1256 m^2^). The number of each plant species in each quadrat was recorded.

### 2.6. Nearest-Neighbor Analysis for Plant Community

Nearest-neighbor analysis was used to examine interactions between the plant roosted on by the largest locust group and other plants according to a modification of the methods of Fonteyn and Mahall [25]. In this method, the distance between the center plant (with the largest locust group) and its nearest neighbor is recorded, as is the sum of the plant sizes of each member of the nearest-neighbor pair. Linear regression of size on distance is then calculated from these measurements. It is postulated that if these two variables are positively correlated, then there is interference between neighboring plants.

### 2.7. Geotaxis Experiments

To examine whether negative (i.e., ascending) geotaxis plays a role in aggregation formation on the night-roosting plants, the locusts’ geotactic response to gravity was investigated at night (21:00–23:00). One hundred sixty nymphs were collected from migratory bands around dusk and kept in a large meshed cage (length × width × height: 100 cm × 50 cm × 50 cm) under natural conditions. The nylon mesh walls allowed nymphs to climb. Ten females and ten males were taken from the cage and released at the bottom of another rectangle meshed cage (50 cm × 50 cm × 100 cm) and allowed to settle for 10 min. The cage was then turned upside down and their distribution was recorded after 10 min (i.e., 20 individuals were tested at the same time). The cage position was divided into three zones: lower (0–30 cm from the base), middle (>30–70 cm from the base), and upper (>70–100 cm from the base). If nymphs were distributed in the upper zone, their geotactic response was regarded as negative; those distributed in the lower zone were regarded as showing positive geotaxis. This test was repeated once for each group, and the experiments were replicated four times. The cage did not turn upside down after locust introduction was used for control.

### 2.8. Statistical Analysis

Tukey–Kramer HSD tests and Steel–Dwass test were conducted to analyze the significance of differences in plant size and locust group size among the four plant species, respectively. Percentages of various group sizes that roosted on each plant species were subjected to a post hoc Fisher’s exact test after Bonferroni correction. ANCOVAs were used to analyze the effects of plant species and size on group size. To analyze the relationship between nearest-neighbor distance and plant size, we used Pearson correlation. A sigmoid curve was fitted between the nearest-neighbor distance and group size. These analyses were conducted using the software packages R (R Development Core Team, Vienna, Austria) [26] and JMP (SAS Institute, Cary, NC, USA).

## 3. Results

### 3.1. Night-Roosting Plant Preference

**Figure 2:**
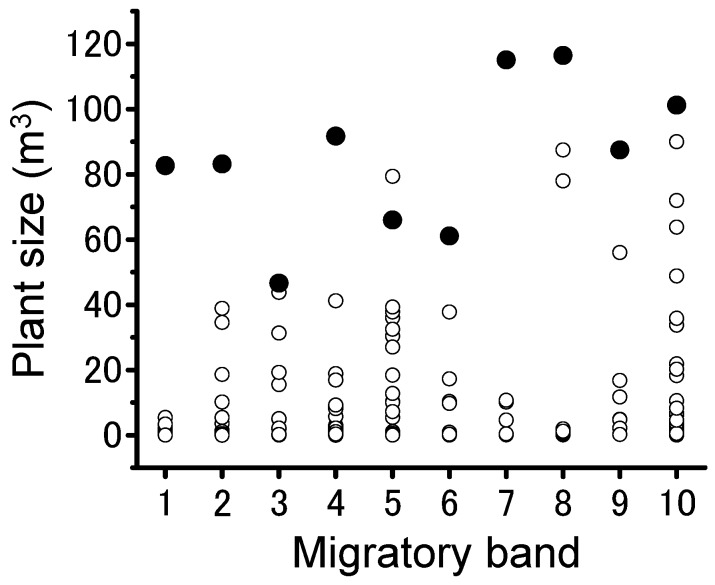
Vertical distribution of plant size (m^3^) at each site where 10 migratory bands of *Schistocerca gregaria* roosted, respectively. Closed circles indicate the plants roosted on by the largest locust group (the center) and open circles indicate other individual plants in the local area (within 20 m from the center). The largest aggregation formed on the largest tree within the plant community in nine of the ten bands.

**Figure 3:**
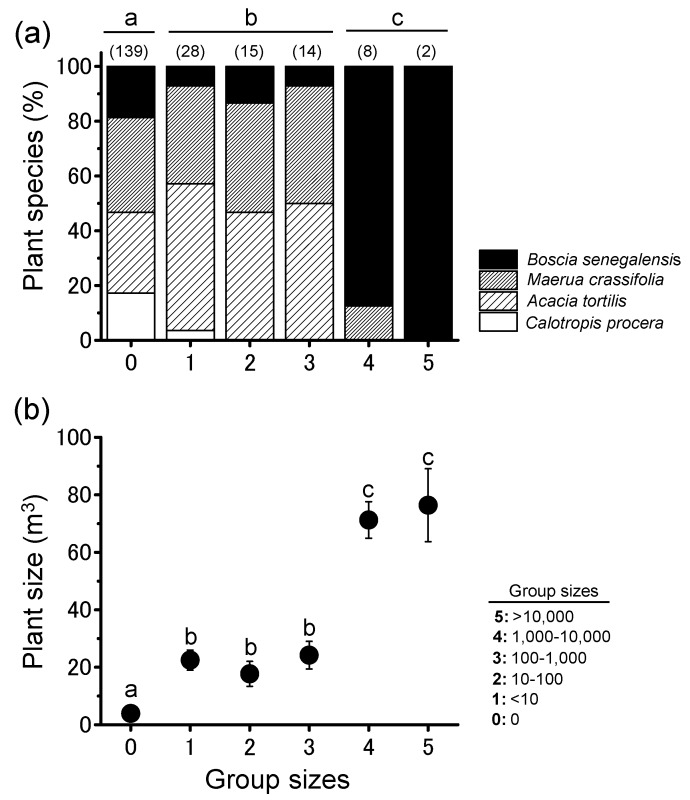
(**a**) Percentage of four plant species; and (**b**) size of plant (m^3^) roosted on by various group sizes of gregarious *Schistocerca gregaria* nymphs at night. Numbers in parentheses are sample sizes. Different letters on the bars indicate significant differences at *p* < 0.016 (post hoc Fisher’s exact test after Bonferroni correction). Different letters above each circle indicate significant differences at *p* < 0.05 (Tukey–Kramer HSD test). Error bars mean SE.

Actively marching migratory bands of gregarious nymphs passed some plants and finally roosted on patchily distributed trees around dusk and aggregated there (Figure 1a–c). At night, locust groups were rarely observed on the ground, grass, or small trees, irrespective of plant species. Nine of the ten migratory bands formed the largest group on the largest tree within the local plant community (Figure 2). They roosted on all four tree species commonly encountered during migration, but they formed the largest groups on *B. senegalensis* and *A. tortilis*. (Figure 3a and Table 1; Fisher’s exact test after Bonferroni correction, *p* < 0.016). Relatively small groups of locusts were found on smaller plants (Figure 3b). As a result, night-roosting plants used by larger groups were significantly larger than those used by smaller groups. Although medium-sized plants may have physical availability of roosting space for the large groups, the migratory bands mainly climbed the largest trees, indicating that they can evaluate plant size from the ground. ANCOVA showed that plant size significantly influenced the group size (Table 2). Although maximum plant width and height showed a significantly positive relationship with group size (Pearson’s correlation, *r* = 0.868, *n* = 206, *p* < 0.001), a more detailed two-way ANOVA of plant characteristics showed that plant height rather than plant width was the key factor influencing group size (Table 3).

### 3.2. Locust Groups’ Interaction with the Plant Community

**Figure 4:**
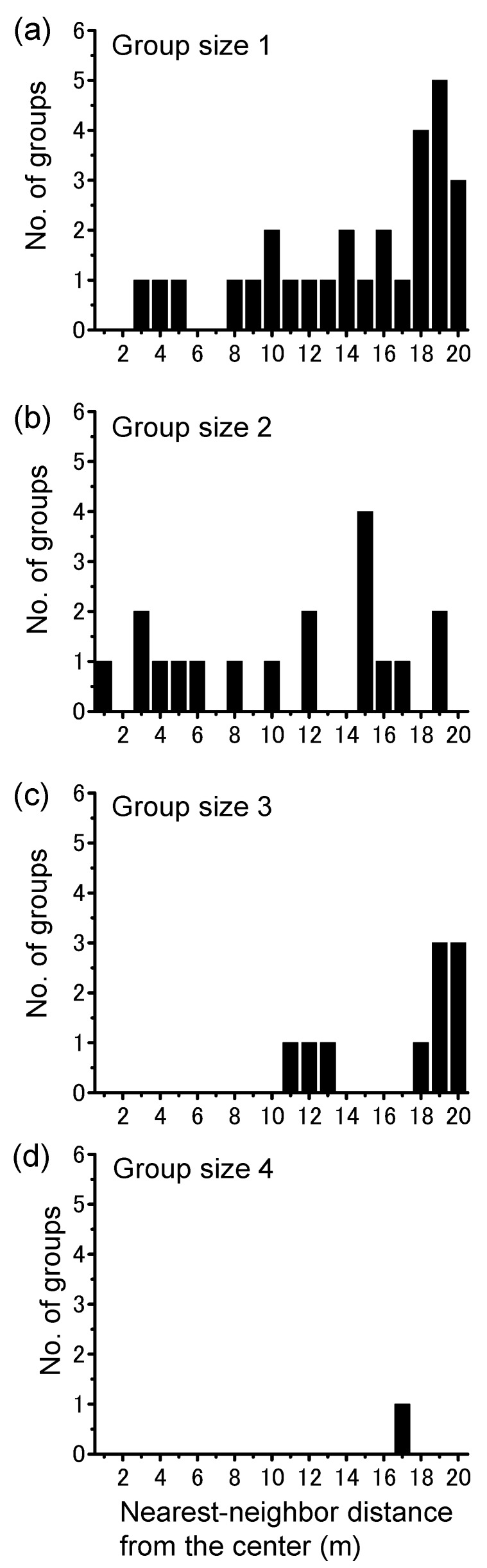
Distribution of various locust group sizes: (**a**) 1 (<10 locusts); (**b**) 2 (10–100); (**c**) 3 (100–1000); and (**d**) 4 (1000–10,000) associated with distance (m) from the center plant containing the largest aggregation. Group size 5 (>10,000 locusts) was not observed. Note that large groups other than the main group were not distributed near the center.

**Figure 5:**
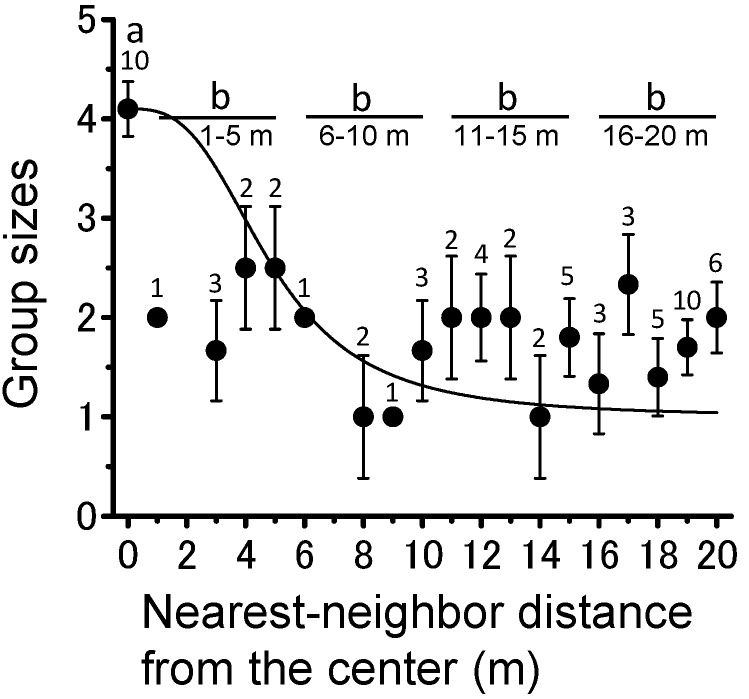
The relationship between trees roosted on by the largest group of gregarious *Schistocerca gregaria* nymphs and other group sizes associated with distance from the center (m). Different letters above circles indicate significant differences in group size based on the sum of every 5 m at *p* < 0.05 (Steel–Dwass test). Numbers above circles are sample sizes. Error bars mean SE. See group sizes in Figure 2.

Theoretically, if migratory bands maintain collective movement when they climb a night-roosting tree, a single large group would be formed with no other large groups scattered in the landscape. Nearest-neighbor distance analysis supported this idea and showed that roosting bands comprised a single large group, with various scattered smaller groups (Figure 4). Smaller groups (group sizes 1 and 2) were widely distributed over the survey areas, but larger groups (group sizes 3 and 4) were not formed near the largest group (Figure 4). The relationship between the nearest-neighbor distance and group size generated a significant sigmoid curve (Figure 5; *r*^2^ = 0.52, χ^2^ = 0.24, *n* = 21, *p* < 0.001), indicating that no corresponding largest group was formed in the local area. No significant differences were observed in group size among the various nearest-neighbor distances except for the center (Figure 5; Steel–Dwass test, *p* > 0.05).

### 3.3. Plant Community

**Figure 6:**
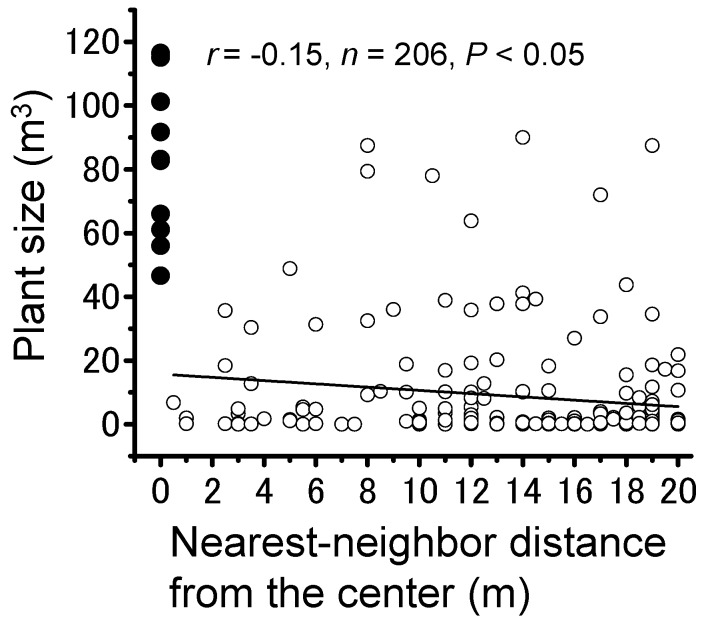
Relationship between distance from the center tree roosted on by the largest group of gregarious *Schistocerca gregaria* nymphs and individual plant size (m^3^).

**Figure 7:**
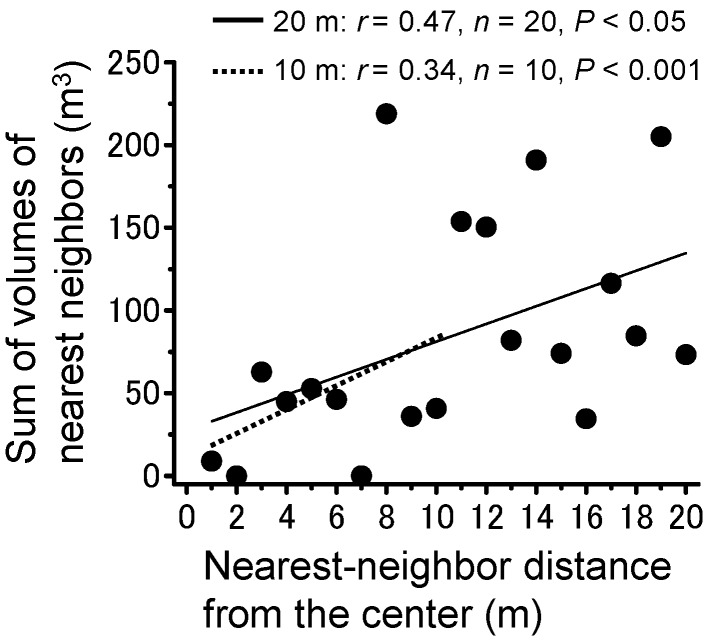
Nearest-neighbor analysis for sum of volumes of individual plants within a local area and distance between nearest neighbor and the center tree where the largest group of roosting locusts aggregated.

To examine the interaction between the locally largest trees where the largest locust group formed and other potential roosting plants, density, and size within the survey area were analyzed (Figure 6). Plant density was nearly constant at each distance (Pearson’s correlation; *r* = −0.37, *n* = 21, *p* > 0.05). Relatively large plants were not distributed near the center, and the plant size slightly decreased as the distance from the center increased (Figure 6; Pearson’s correlation; *r* = −0.15, *n* = 206, *p* < 0.001). Nearest-neighbor analysis for the sum of volumes of individual plants and its nearest neighbor showed a significantly positive correlation at both a long range (20 m, Figure 7; Pearson’s correlation; *r* = 0.47, *n* = 21, *p* < 0.05), indicating interference between the plants, and at a short range (10 m; Pearson’s correlation; *r* = 0.34, *n* = 11, *p* < 0.001). These results suggested that the trees roosted on by the largest locust group were conspicuous within the local plant community.

### 3.4. Geotaxis Experiments

To examine whether locust nymphs oriented toward the tops of trees after sunset, their geotactic response was examined at night. All nymphs synchronously responded to the test cage being turned upside down by climbing the wall and remained in the upper zone and immobile (100%, *n* = 80). Locusts remained upper zone if their cage did not turn upside down. This result indicated that gregarious nymphs show a negative geotactic response at night.

## 4. Discussion

The present study found that more gregarious nymphs aggregated on the larger plants within the local plant community as a night-roosting site during migration. Kennedy suggested that gregarious nymphs were attracted to larger plants [14]. Our observations suggested that plant size (height) was the primary cue used by gregarious nymphs for night-roosting site choice. Plant height seems to be a useful criterion for ground-dwelling locust nymphs because they can visually recognize it from all directions and it is comparable and available in all their habitats.

In the desert plant community, resource competition for water results in a heterogeneous patchy distribution at certain sites [25], as was observed in the present survey area. Our results also suggested that the largest trees in a local area were conspicuous, because no other large plants existed near them. This may allow gregarious nymphs to see the roosting tree from afar without the need to evaluate other potential roosting plants. The simple criterion of plant size seems to be useful for migratory locusts as they encounter various habitats characterized by different vegetation cover, plant species, and plant size [27]. Where landmark trees were absent, we previously observed that migratory bands roosted on relatively small bushes (<1 m height [24]), but they were not used by locusts in the present study. Migratory bands also roosted behind the stones in the bottom side and next to the dunes at its down where trees were absent and bushes were rare (M.A. Ould Babah Ebbe, personal observation). Therefore, such flexible night-roosting site using seems to be adaptive for migratory arthropods to aggregate at the optimal roosting site within a local area. In fact, plant size is frequently used by insects as a cue determining aggregation site, known as “hill-topping” behavior [28]. Hill-topping is mainly reported in flying insect species and rarely observed in flightless ones due to their lower mobility.

To aggregate with conspecifics during a restricted time, synchronous decision making is required for group members. In gregarious nymphs of *S. gregaria*, night-roosting site searching began around dusk [12]. Band members synchronously climbed plants around dusk (17:30–19:00). Some short-range aggregation pheromones have been reported in *S. gregaria* [29], but long-range ones are still unclear. Because night-roosting site choice may be visual and nymphs did not frequently change the roosting trees after sunset, they need to reach the appropriate site by sunset. The timing of climbing may vary depending on the time of day, weather conditions, and availability of sufficiently large plants. In the Sahara Desert, environmental factors fluctuate unexpectedly, but decreasing temperature and light intensity may be reliable signals of dusk. Recently, we found that gregarious nymphs climbed night-roosting plants after fully feeding, which indicates that hunger level is also related to initiation of night-roost searching (O.K. Maeno, personal observation). Previous and present field studies observed that locusts show unclearly oriented wandering behaviors around dusk [12,14,30]. Sometimes this aimless wandering was observed when marching migratory bands walked on open ground without plants. The search for night-roosting plants may be strongly related to this type of marching. Therefore, members of migratory bands may synchronously integrate various information including the relative plant size, time of day, light conditions, individual attraction and physiological conditions (hunger level) when choosing roosting plants.

Night-roosting aggregation on large plants probably functions as an anti-predatory defense in grasshoppers [31,32]. The ground may be a dangerous place for locusts at night, because nocturnal ground predators such as insectivorous jerboa (*Jaculus jaculus*, Dipodidae) and desert hedgehog (*Paraechinus aethiopicus*, Erinaceidae) hunt on the ground [33]. We observed very few locusts on the ground at night, and roosting high in trees allows locust nymphs to escape nocturnal ground predators. In the present survey site, some smaller plants had enough space for many nymphs to roost, but the locusts avoided using these plants, probably because they did not provide functional shelter. Although some nocturnal predators, such as camel spiders, do hunt in trees, they are solitary arthropods that are likely quickly satiated. Aggregation at night may reduce predation risk through the dilution effect [3,7]. Forming a “selfish herd” seems to be critical for night-roosting locusts, because their movement is constrained by low temperature and darkness at night. In the present survey area, ambient temperature can fall below 20 °C at night. Because escaping performance is temperature-dependent in grasshoppers [34], gregarious nymphs cannot escape quickly from approaching predators at night. At low temperature, prey ectotherms frequently use refuges to protect themselves from predators [35,36]. If immobile conspecifics aggregate with each other at a limited site, individual locusts may protect themselves from predators via satiation. Similar anti-predatory benefits by aggregating with immobile conspecifics at night was reported in the bee *Idiomelissodes duplocincta* [37]. Sword et al. confirmed this shellfish herd in Mormon crickets, *Anabrus simplex,* and demonstrated that individual predation risk was lower in crowd than not within crowd [38]. Thus, aggregation of gregarious nymphs within a limited area in protective refuge plants seems to be adaptive to increase the chance of survival during a vulnerable period.

Migratory bands formed various sized groups on patchily distributed plants during a night. Spatial distribution analyses showed that migratory bands formed one large group and several scattered relatively smaller groups at the local level. As described above, some scattered small marching bands were also attracted to the large roosting tree, meaning that scattered bands can fuse to form an aggregation via night-roosting choice. If predators encounter prey animals, they frequently switch from random to concentrated searching [39]. When predators detect the single large group, however, they soon become satiated. Forming a large aggregation within a restricted area, rather than widely scattered smaller aggregations, would also reduce the probability of predation. Thus, predation pressure has likely driven night-roosting site choice and aggregation formation in *S. gregaria*.

## 5. Conclusions

The desert locust is a notorious pest causing serious losses to agricultural crops. Understanding the species-, phase- and developmental-specific microhabitat use pattern can provide not only critical insight into pest management techniques, such as trapping and mass control, but also understanding the mechanism underlying gregarization [10]. Spraying all individuals scattered within an entire infested zone is arguably both financially and environmentally unacceptable [40]. The present study found the importance of large plants as a potential aggregation site. Identifying the preferred plant characteristics is essential for developing artificial trapping systems for locusts and inciting new night control approaches that are more efficient and environmentally friendly.

## Figures and Tables

**Table 1:** Mean (±SE) number of plants per circular quadrat (40 m diameter, *n* = 10) and plant size of four species frequently roosted on at the survey site.

Plant Species	No. of Plants (1256 m^2^)	No. of Quadrads		Plant		No. of Plants
			Maximum Width (m)	Maximum Height (m)	Volume (m^3^)	
*Calotropis procera*	3.4 ± 3.0	10	0.97 ± 0.25 ^a^	1.03 ± 0.23 ^ab^	2.7 ± 4.3 ^a^	25
*Acacia tortilis*	7.1 ± 3.0	10	1.17 ± 0.15 ^a^	0.91 ± 0.14 ^a^	9.1 ± 2.0 ^a^	71
*Maerua crassifolia*	6.1 ± 3.0	10	2.14 ± 0.15 ^b^	2.34 ± 0.14 ^c^	21.8 ± 3.1 ^b^	70
*Boscia senegalensis*	4.1 ± 3.0	10	2.04 ± 0.19 ^b^	1.67 ± 0.18 ^b^	30.4 ± 4.8 ^b^	40

Different letters after values indicates significant differences between values within a column (Tukey–Kramer test, *p* < 0.05).

**Table 2:** Three-way analysis of variance for group size of gregarious *Schistocerca gregaria* nymphs on night-roosting plants.

Source of Variance	Group Size on Roosting Plants			
	*df*	*ms*	*f*	*p*
plant species	3	0.94	1.12	0.291
plant size	1	16.67	19.83	<0.001
distance from the center	1	0.63	0.75	0.389
plant species × plant size	1	0.29	0.34	0.559
plant species × distance	1	0.89	1.05	0.306
plant size × distance	1	0.22	0.27	0.607
plant species × plant size × distance	1	3.21	3.82	0.052
error	198	166.45		

**Table 3:** Two-way analysis of variance for group size of gregarious nymphs of *Schistocerca gregaria* considering the influence of plant width and height.

Source of Variance		Group Size on Roosting Plants		
	*df*	*ms*	*f*	*p*
width	1	0.1327117	0.1695	0.681
height	1	8.961103	11.4446	0.0009
width × height	1	0.3010408	0.3845	0.5359
error	198	166.45		
